# Radiation therapy alleviates Doege–Potter syndrome accompanied with severe aortic valve insufficiency: a case report and literature review

**DOI:** 10.3389/fonc.2025.1681861

**Published:** 2025-11-24

**Authors:** Guiliang Peng, Rongwan Sun, Xiaotian Lei, Chunni Deng, Weiling Leng, Xing Zhang, Youli Wu, Laiping Xie, Pian Hong, Min Long, Liu Chen

**Affiliations:** 1 Department of Endocrinology, The First Affliated Hospital (Southwest Hospital) of Army Medical University, Chongqing, China; 2 Department of Endocrinology, The Chongqing Thirteenth People’s Hospital, Chongqing, China; 3 Department of Pathology, The First Affliated Hospital (Southwest Hospital) of Army Medical University, Chongqing, China; 4 Department of Nuclear Medicine, The First Affliated Hospital (Southwest Hospital) of Army Medical University, Chongqing, China; 5 Department of Outpatient, The 903rd Hospital of the Chinese People’s Liberation Army, Hangzhou, China

**Keywords:** Doege-Potter Syndrome (DPS), non-islet cell tumor hypoglycemia (NICTH), solitary fibrous tumor (SFT), radiation therapy, severe aortic valve insufficiency

## Abstract

**Background:**

Doege–Potter syndrome (DPS) is a rare paraneoplastic condition characterized by hypoglycemia resulting from the excessive secretion of insulin-like growth factor-2 (IGF-2) by a solitary fibrous tumor (SFT). This report presents an elderly patient with DPS complicated by severe cardiac conduction abnormalities, illustrating the clinical manifestation, therapeutic intervention, and multidisciplinary management strategy.

**Case presentation:**

A 78-year-old woman was diagnosed with DPS, presenting with loss of consciousness with a blood glucose level of 1.41 mmol/L, and hypoglycemia was resolved with intravenous glucose. The initial laboratory investigation revealed elevated insulin levels of 7.17 μIU/mL, with suppressed C-peptide levels of 0.09 ng/mL and insulin-like growth factor-1 (IGF-1) levels of 26.47 ng/mL. Thoracic computed tomography (CT) identified a 13.6 cm × 8.6 cm mass in the right lower thoracic cavity. CT-guided transthoracic biopsy confirmed SFT with immunohistochemical positivity for CD34 and STAT6. Electrocardiogram (ECG) demonstrated frequent atrial premature complexes (29,292/24 hours), short runs of atrial tachycardia (144/24 hours), and paroxysmal ventricular premature complexes. Echocardiography revealed severe aortic valve insufficiency. Following multidisciplinary team (MDT) consultation, the surgically ineligible patient received tumor-directed radiotherapy (60 Gy in 30 fractions), glucocorticoid replacement (hydrocortisone 40 mg daily), and overnight carbohydrate supplementation to alleviate hypoglycemia. At 18-month follow-up, serial chest CT showed tumor size reduction. Holter monitoring revealed a substantial reduction in atrial premature complexes (1,955/24 hours vs. baseline 29,292/24 hours). The patient exhibited no recurrence of hypoglycemic episodes.

**Conclusion:**

This report describes a case of DPS with refractory hypoglycemia complicated by severe cardiac structural and conduction abnormalities. Radiotherapy combined with endocrine intervention effectively controlled tumor-associated hypoglycemia in this surgically ineligible patient.

## Introduction

Doege–Potter syndrome (DPS) is a rare paraneoplastic syndrome characterized by severe hypoglycemia resulting from the non-islet cell tumors secreting insulin-like growth factor-2 (IGF-2) or its high-molecular-weight precursor big IGF-2 (1). Doege reported the initial association between solitary fibrous tumor (SFT) and hypoglycemia in 1930 (2). With a prevalence under 5%, DPS typically occurs in large pleural or peritoneal tumors and manifests as fasting hypoglycemia, consciousness impairment, and autonomic symptoms (3). Complete surgical resection remains the definitive treatment (4, 5); however, therapeutic alternatives are limited for patients with comorbid cardiopulmonary conditions contraindicating surgery. Although radiotherapy is primarily utilized for adjuvant treatment or metastasis control, its efficacy as monotherapy for inoperable DPS lacks substantive evidence. Notably, no previous reports have documented DPS coexisting with severe cardiac structural and conduction abnormalities such as severe aortic valve insufficiency. We report the first case of an older woman with DPS complicated by hypertensive heart disease, severe aortic valve insufficiency, and arrhythmia who was deemed surgically ineligible. Following evaluation by the multidisciplinary team (MDT) for rare diseases, she underwent precision low-dose radiotherapy, achieving complete resolution of hypoglycemic symptoms.

## Case presentation

**Figure 1:**
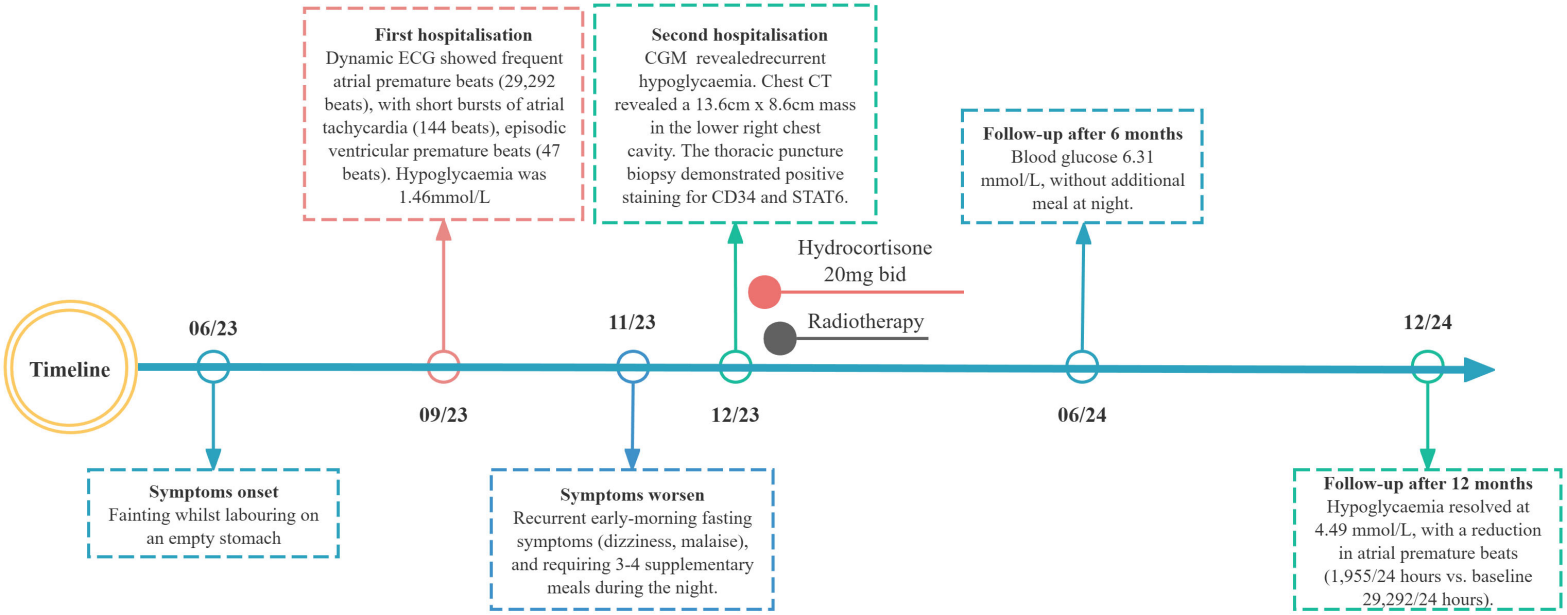
Longitudinal clinical timeline of paraneoplastic hypoglycemia in the patient with thoracic solitary fibrous tumor.

A 78-year-old woman with recurrent fasting syncope was admitted to the First Affiliated Hospital of Army Medical University on December 1, 2023 (**Figure 1**). She suffered from five syncopal episodes in the past 6 months, each lasting seconds to minutes, and accompanied with transient visual obscurations, generalized weakness, diaphoresis, and spontaneous recovery of consciousness upon supine positioning. Prior evaluation in the cardiology unit in September 2023 for recurrent syncope and falls revealed fasting hypoglycemia (glucose, 1.4 mmol/L; postprandial normalization to 4.6 mmol/L). Echocardiography demonstrated left ventricular dilatation (end-diastolic diameter, 58 mm), mild basal interventricular septal hypertrophy (12.5 mm), severe aortic regurgitation, and mild mitral regurgitation. Dynamic electrocardiogram (ECG) showed sinus rhythm (mean heart rate, 80 beats per minute), frequent atrial premature complexes (29,292), short runs of atrial tachycardia (144), occasional ventricular premature complexes (47), junctional escape beats, and reduced heart rate variability (Standard Deviation of Normal-to-normal intervals (SDNN), 80 ms; Standard Deviation of the Average Normal-to-normal intervals (SDANN), 60 ms; triangular index, 23.4). Post-discharge, she experienced recurrent early-morning fasting symptoms, characterized by dizziness and general discomfort relieved by carbohydrate ingestion (e.g., glucose and sesame paste). However, blood glucose was not monitored. Since November 5, 2023, she has experienced a progressive decline in physical capability, with difficulty waking up in the morning, unresponsiveness, and mild-to-moderate exertional limitation, and has been requiring three to four nocturnal supplemental meals for symptom relief. Past medical history included hypertensive heart disease, severe aortic valve insufficiency, and arrhythmia. Current medications were sacubitril valsartan sodium (100 mg twice daily), nifedipine (30 mg daily), and atorvastatin (20 mg daily). No significant personal or family history was reported.

Upon admission, the patient had a temperature of 36.2°C, a respiratory rate of 20 breaths per minute, a heart rate of 116 beats per minute, a blood pressure reading of 155/98 mmHg, a height of 148 cm, a weight of 62 kg, and a body mass index of 28.3 kg/m^2^. Physical examination revealed abdominal obesity, perioral cyanosis, and a diastolic murmur over the aortic area. No evidence of arrhythmia was observed. Furthermore, there was an absence of indications suggestive of acromegaly.

On December 2, 2023, at 02:00, the patient experienced loss of consciousness. Continuous glucose monitoring (CGM) recorded a blood glucose level of 1.6 mmol/L, confirmed by venous measurement of 1.33 mmol/L. Intravenous administration of 40 mL of 50% dextrose raised blood glucose to 3.1 mmol/L by 02:35, and 20 mL dextrose maintained glucose at 7.0 mmol/L by 03:05. Laboratory evaluation revealed insulin at 7.17 μIU/mL (reference range, 5–25 μIU/mL), C-peptide at 0.09 ng/mL (0.25–4.0 ng/mL), insulin release index of 0.3, β-hydroxybutyrate at 0.4 mmol/L, cortisol at 307.95 nmol/L, growth hormone at 0.64 ng/mL, gastrin-releasing peptide precursor at 63.27 pg/mL, gastrin G-17 at 1.90 pmol/L, and hemoglobin A1c of 5.6%. An oral glucose tolerance test (OGTT) revealed fasting blood glucose of 3.41 mmol/L, insulin of 10.07 μIU/mL, and C-peptide of 0.81 ng/mL; 2-hour postprandial values were 12.08 mmol/L for glucose, 28.98 μIU/mL for insulin, and 1.53 ng/mL for C-peptide, consistent with impaired glucose tolerance. Acarbose 50 mg was initiated to delay carbohydrate absorption. During hospitalization, recurrent spontaneous hypoglycemia occurred and was consistently relieved by oral carbohydrate intake. Additional endocrine evaluations revealed growth hormone at 0.52 ng/mL, IGF-1 at 26.47 ng/mL (78.7–226 ng/mL), IGF-2 at 107.71 ng/mL, IGF-2/IGF-1 ratio of 4.55, and insulin-like growth factor binding protein-3 (IGFBP-3) at 2,207 ng/mL; adrenocorticotropic hormone (ACTH) at 13.23 pg/mL (3.1–6.8 pg/mL) with cortisol at 530.69 nmol/L (181.83–787.93 nmol/L); and free triiodothyronine at 4.77 pmol/L (3.1–6.8 pmol/L), free thyroxine at 13.0 pmol/L (12–22 pmol/L), and thyroid-stimulating hormone at 1.56 μIU/mL (0.27–4.2 μIU/mL). Parathyroid hormone, estradiol, progesterone, follicle-stimulating hormone, luteinizing hormone, liver function tests, renal function, and lipid profile were unremarkable. Insulin antibody (IAA), islet cell antibody (ICA), and glutamic acid decarboxylase antibody (GAD) were negative.

**Figure 2:**
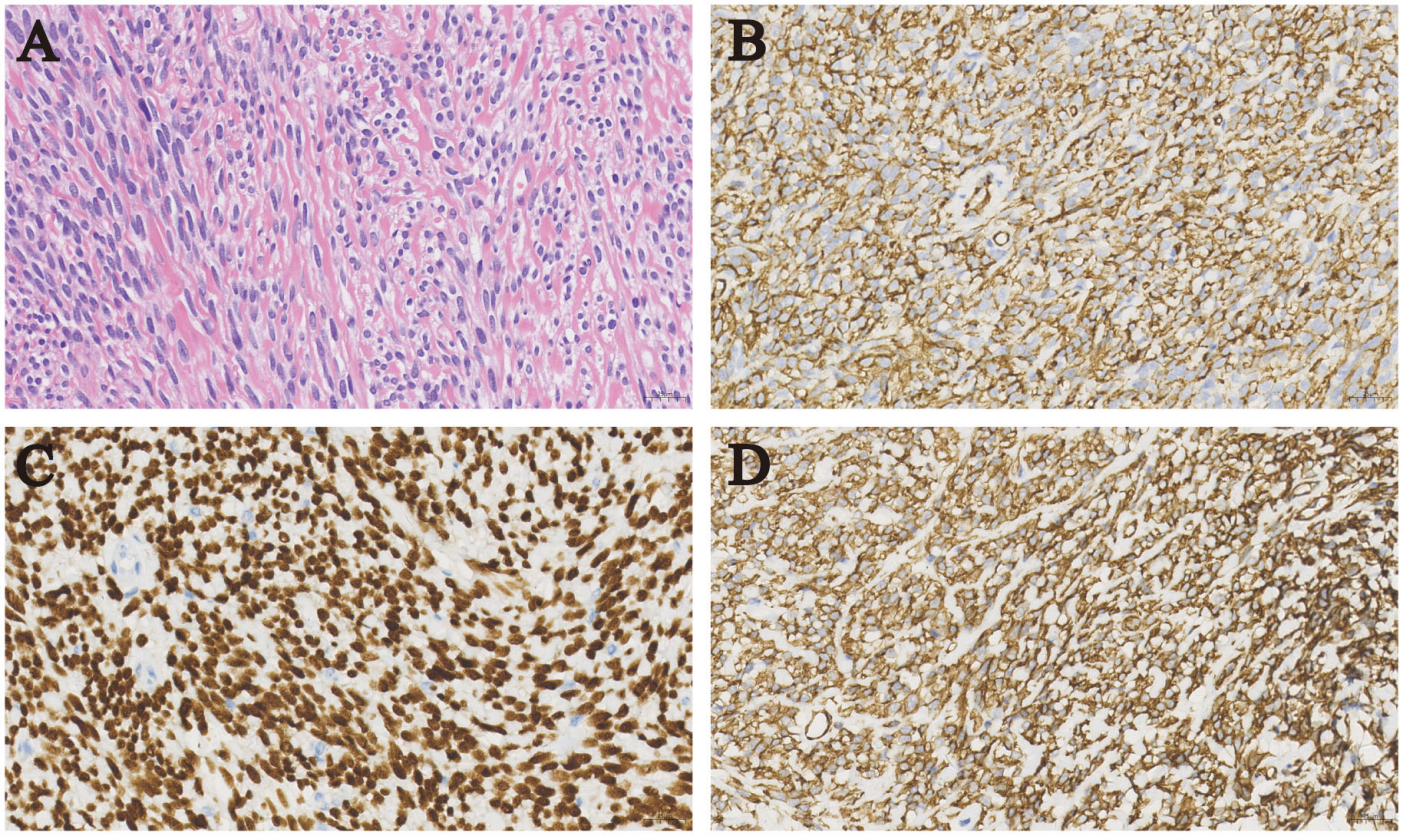
Histopathology of solitary fibrous tumor. Hematoxylin and eosin staining showed enlarged spindle cells with a variable proportion of collagenous stroma **(A)**. Positive immunohistochemical staining for CD34 **(B)**, STAT6 **(C)** and Vim **(D)**, original magnification×400.

**Figure 3:**
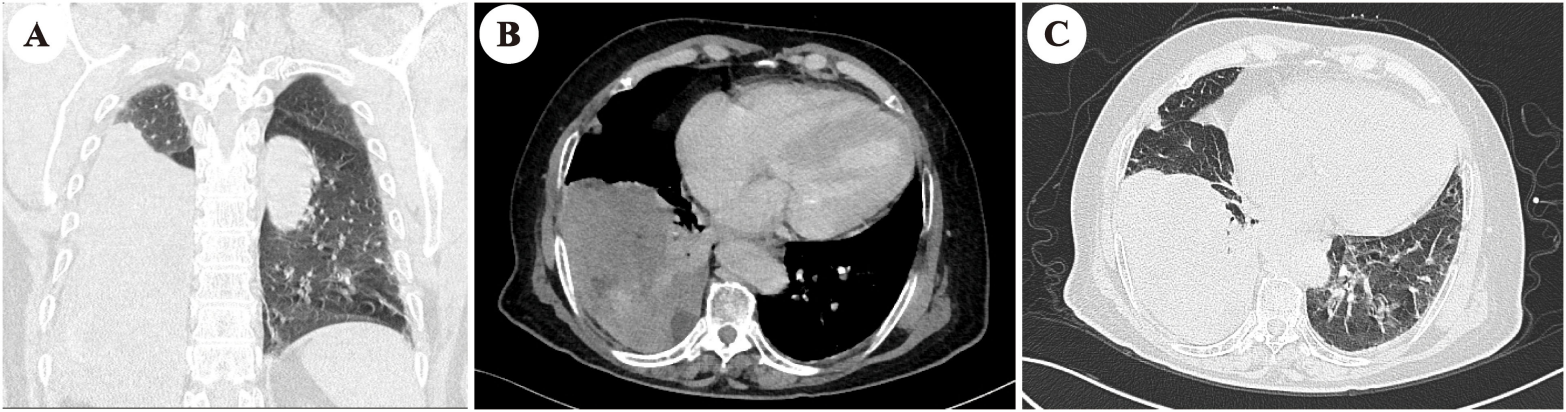
The chest CT revealed a giant heterogeneous mass in the right lower thoracic cavity (13 cm × 8.6 cm). **(A)** Coronal, maximum intensity projection CT images of the chest. **(B)** Sagittal portal phase imaging. **(C)** Sagittal arterial phase images.

Abdominal contrast-enhanced CT demonstrated normal morphology and density of the liver, kidneys, and pancreas. Chest CT indicated a 13.6 × 8.6 cm mass in the right lower thorax with heterogeneous density, calcification, and adjacent lung lobe compression (**Figure 2**). MRI of the sella region revealed a 4.6-mm pituitary microadenoma. Pathologic examination of the thoracic mass biopsy showed positive immunohistochemical staining for vimentin, desmin, CD34, STAT6, p53, and β-catenin, with a Ki-67 proliferation index of 3% (**Figure 3**), confirming SFT.

The elderly patient had severe cardiac valvulopathy and conduction system disease, with a surgical risk EuroSCORE II of 12.5%. Following MDT consultation, radiotherapy was initiated (60 Gy in 30 fractions), and hydrocortisone (40 mg daily) and carbohydrate supplementation were prescribed in an attempt to prevent hypoglycemia. At follow-up, the patient discontinued the use of hydrocortisone without medical supervision. However, the patient reported reduced hypoglycemia frequency, with nocturnal carbohydrate intake only once or twice weekly. Six months post-radiotherapy, nocturnal supplementation was discontinued; blood glucose stabilized at 6.31 mmol/L. Laboratory studies showed persistently low IGF-1 and IGFBP-3 levels, with IGF-2 elevated but IGF-2/IGF-1 ratio <3.0. At 12 months, the patient remained asymptomatic with fasting blood glucose of 4.49 mmol/L and persistent low IGF-1 and IGFBP-3 (**Table 1**). Repeat chest CT demonstrated a stable right lower thoracic mass (11.8 × 8.5 cm). Ambulatory ECG monitoring revealed sinus rhythm with frequent atrial premature beats (1,955 beats) and ST-T changes.

**Table 1:** Follow-up characteristics of case laboratory parameters.

Time	Glu (mmol/L)	GH (ng/mL)	IGF-1 (ng/mL)	IGF-2 (ng/mL)	C-p (ng/mL)	INS (μIU/mL)	CORT (nmol/L)	ACTH (pg/mL)
Hospitalization	1.33	0.64	26.47	–	0.09	7.17	530.69	60.14
01/16/2024 (HC+RT+AM)	3.55	0.25	23.68	107.71	3.12	16.84	546.05	4.5
02/22/2024	5.21	1.09	32.51	126.97	3.12	16.84	391.72	37.46
03/10/2024	5.75	3.1	47.3	80.98	5.55	35.46	510.96	16.93
06/15/2024 (No AM)	6.31	1.46	34.09	127.59	–	29.91	601.09	25.2
12/12/2024	4.49	1.29	40.79	–	4.09	21.46	108.84	5.06

AM, additional meals; HC, hydrocortisone; RT, radiotherapy.

Hormones (range): adrenocorticotropic hormone (ACTH; 5–60 pg/mL), cortisol (CORT; 181.83–787.93 nmol/L), C peptide (C-p; 0.25–4.0 ng/mL), growth hormone (GH; 0–5 ng/L), glucose (Glu; 3.9–6.1 mmol/L), insulin (INS; 5–25 μIU/mL), and insulin-like growth factor 1 (IGF-1; 78.7–226 ng/mL).

## Discussion

Doege–Potter syndrome is a paraneoplastic syndrome, most prevalent in individuals aged 50–70 years without gender predilection (6). It typically manifests as hypoglycemia due to IGF-2-mediated suppression of insulin secretion, and the diagnosis requires fulfillment of Whipple’s triad (7, 8): 1) symptoms consistent with hypoglycemia, 2) documented plasma glucose ≤2.8 mmol/L during symptoms, and 3) symptom resolution following glucose normalization. In this case, recurrent syncope with hypersomnolence, diaphoresis, and fatigue occurred exclusively during fasting states (morning or nocturnal) and resolved with carbohydrate intake, consistent with non-islet cell tumor hypoglycemia (NICTH) (9). However, concomitant arrhythmia and severe aortic valve insufficiency complicated the attribution of syncope etiology due to overlapping cardiovascular and hypoglycemic pathologies. Hypoglycemia induces myocardial ischemia and hypoxia, impairing regular electrophysiological activity and exacerbating conduction system delays or blocks. Critically, the profound hypoglycemia (1.33 mmol/L) observed during admission lacked correlation with bedside ECG findings, precluding confirmation of a causal link to arrhythmias. Following the correction of hypoglycemia per therapeutic protocol, syncope resolved without recurrence, indicating that recurrent hypoglycemia was the primary underlying cause.

Characteristic biochemical features of NICTH include suppressed insulin, C-peptide, and β-hydroxybutyrate levels. However, C-peptide and insulin assays are often omitted during initial DPS evaluations (10, 11). Laboratory findings in this patient confirmed no diabetes medication use, with suppressed insulin (7.17 μIU/mL) and C-peptide (0.09 ng/mL) levels, consistent with NICTH. Although the OGTT demonstrated impaired glucose tolerance (fasting glucose, 3.41 mmol/L; 2-hour, 12.08 mmol/L), nocturnal hypoglycemia requiring intravenous dextrose invalidated the test and precluded a 72-hour fast. Negative insulin autoantibodies and routine pancreatic imaging excluded insulinoma and autoimmune hypoglycemia. Paradoxically, despite hypoglycemia typically stimulating counter-regulatory hormones such as cortisol, GH, and IGF-1 (12), this patient exhibited subnormal cortisol (307.95 nmol/L) with elevated ACTH (60.14 pg/mL), suppressed GH (0.52 ng/mL), and IGF-1 (26.47 ng/mL). This dissociation may reflect chronic hypoglycemia-induced suppression of counter-regulatory axes (13), with persistent cortisol impairment despite pituitary microadenomas (10). Definitive diagnosis of DPS as a paraneoplastic manifestation of SFT requires radiographic localization and immunohistochemical confirmation of CD34 and STAT6 expression.

Complete surgical resection is the standard treatment for DPS. However, in patients with contraindications to surgery, such as severe cardiac comorbidities or those with unresectable tumors, hypoglycemia may persist despite apparent complete resection (14–17). Conventional medical therapies for glycemic control demonstrate limited efficacy in NICTH: intravenous glucose provides only transient relief, and glucocorticoids, glucagon, GH, or somatostatin analogs often fail to suppress IGF-2-mediated hypoglycemia (18–25). In this case, the patient failed to achieve improvement in hypoglycemic episodes following treatment with hydrocortisone and acarbose. Although chemotherapy (e.g., doxorubicin) or anti-angiogenic agents may reduce tumor burden and ameliorate hypoglycemia (26, 27), robust evidence for their use in DPS remains scarce.

Radiotherapy, as a non-invasive means, may inhibit tumor IGF-2 secretion by local irradiation and could be an innovative therapeutic option for inoperable DPS. In this 78-year-old patient, palliative radiotherapy (60 Gy in 30 fractions) targeting the SFT normalized the IGF-2/IGF-1 ratio, with sustained euglycemia (fasting glucose, 4.49 mmol/L) and discontinuation of nocturnal carbohydrate supplementation. This suggests that radiotherapy may induce tumor cell death and suppress IGF-2 secretion without requiring significant tumor shrinkage, as evidenced by stable tumor dimensions on follow-up CT. Notably, the efficacy of radiotherapy in NICTH remains controversial across reports: some describe persistent hypoglycemia despite treatment (28, 29), while others document partial or complete resolution. This case supports the latter pattern. Furthermore, radionuclide therapies have shown preliminary success in complex cases, such as severe hypoglycemia with solitary fibrous tumors or malignant insulinomas (30, 31), highlighting the potential of targeted internal radiation in select patients. Notably, the resolution of prolonged RR intervals (>2.0 seconds) on ambulatory ECG paralleled hypoglycemia correction, indicating that autonomic dysfunction in NICTH may be reversible with metabolic stabilization. Prolonged hypoglycemia in this patient caused not only substantial physiological distress but also profound psychological burdens, including anxiety and helplessness. The application of a non-conventional therapeutic approach successfully alleviated her symptoms and, more importantly, restored her hope for a meaningful life.

## Conclusions

This report describes a notable case of DPS in a 78-year-old woman complicated by severe aortic valve insufficiency and arrhythmia. Despite being treated with a combination of acarbose and hydrocortisone, the patient continued to suffer from refractory hypoglycemia. After a consensus from the MDT, palliative radiotherapy was initiated. At 12-month follow-up, fasting plasma glucose levels had stabilized within the normal range, and episodes of hypoglycemia had completely resolved.

## Data Availability

The raw data supporting the conclusions of this article will be made available by the authors, without undue reservation.
